# Bis(dimethyl sulfoxide-κ*O*)tetra­kis­(μ_2_-3,4,5-tri­meth­oxy­benzoato-κ^2^ *O*:*O*′)dizinc

**DOI:** 10.1107/S1600536813023118

**Published:** 2013-08-23

**Authors:** Diana Deutsch, Thomas Zevaco, Olaf Walter

**Affiliations:** a IKFT, KIT-Campus Nord, Hermann-von-Helmholtz-Platz 1, 76344 Eggenstein-Leopoldshafen, Germany

## Abstract

The colourless title complex, [Zn_2_(C_10_H_11_O_5_)_4_(C_2_H_6_OS)_2_], crystallizes with one half-mol­ecule in the asymmetric unit, the other half of the mol­ecule being generated by a crystallographic inversion center. The structure shows a μ_2_-*O*:*O*′-bridging mode of the four 3,4,5-tri­meth­oxy­benzoate ligands finally stabilizing the two Zn^II^ atoms in the dinuclear complex in a distorted square-pyramidal environment. The fifth coord­in­ation site in the apical position of the pyramid is occupied by a coordinating dimethyl sulfoxide solvent mol­ecule equally disordered over two positions.

## Related literature

For the structures of (μ_2_-benzoato-κ*O*,*O*′)(di­methyl­sulfoxide-κ*O*)dizinc complxes with no more additional ligands, see: Pham *et al.* (2008 ▶); Reger *et al.* (2011 ▶); Tao (2002 ▶); Yang *et al.* (2005 ▶); Zevaco *et al.* (2007 ▶).
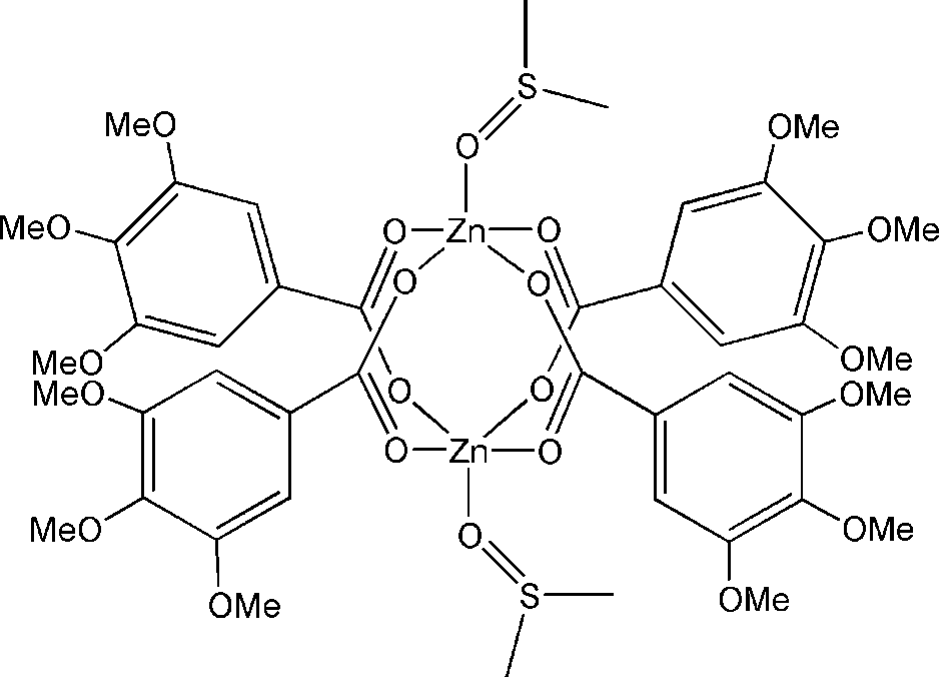


## Experimental

### 

#### Crystal data

[Zn_2_(C_10_H_11_O_5_)_4_(C_2_H_6_OS)_2_]
*M* *_r_* = 1131.75
Monoclinic, 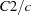

*a* = 18.854 (2) Å
*b* = 13.937 (2) Å
*c* = 19.249 (2) Å
β = 90.082 (3)°
*V* = 5058.0 (11) Å^3^
*Z* = 4
Mo *K*α radiation
μ = 1.11 mm^−1^
*T* = 200 K
0.6 × 0.4 × 0.4 mm

#### Data collection

Siemens SMART CCD 1000 diffractometer
Absorption correction: multi-scan (*SADABS*; Bruker, 1997 ▶) *T*_min_ = 0.555, *T*_max_ = 1
29984 measured reflections
6234 independent reflections
4049 reflections with *I* > 2σ(*I*)
*R*_int_ = 0.121

#### Refinement

*R*[*F* ^2^ > 2σ(*F* ^2^)] = 0.065
*wR*(*F* ^2^) = 0.164
*S* = 1.03
6234 reflections
378 parameters
19 restraints
H atoms treated by a mixture of independent and constrained refinement
Δρ_max_ = 0.64 e Å^−3^
Δρ_min_ = −0.79 e Å^−3^

### 

Data collection: *SMART* (Bruker, 1997 ▶); cell refinement: *SAINT* (Bruker, 1997 ▶); data reduction: *SAINT*; program(s) used to solve structure: *SHELXS97* (Sheldrick, 2008 ▶); program(s) used to refine structure: *SHELXL2013* (Sheldrick, 2008 ▶); molecular graphics: *XPMA* (Zsolnai, 1996 ▶) and *ORTEP-3 for Windows* (Farrugia, 2012 ▶); software used to prepare material for publication: *publCIF* (Westrip, 2010 ▶).

## Supplementary Material

Crystal structure: contains datablock(s) I. DOI: 10.1107/S1600536813023118/kj2227sup1.cif

Structure factors: contains datablock(s) I. DOI: 10.1107/S1600536813023118/kj2227Isup2.hkl

Supplementary material file. DOI: 10.1107/S1600536813023118/kj2227Isup3.mol

Additional supplementary materials:  crystallographic information; 3D view; checkCIF report

## Figures and Tables

**Fig. 1.**
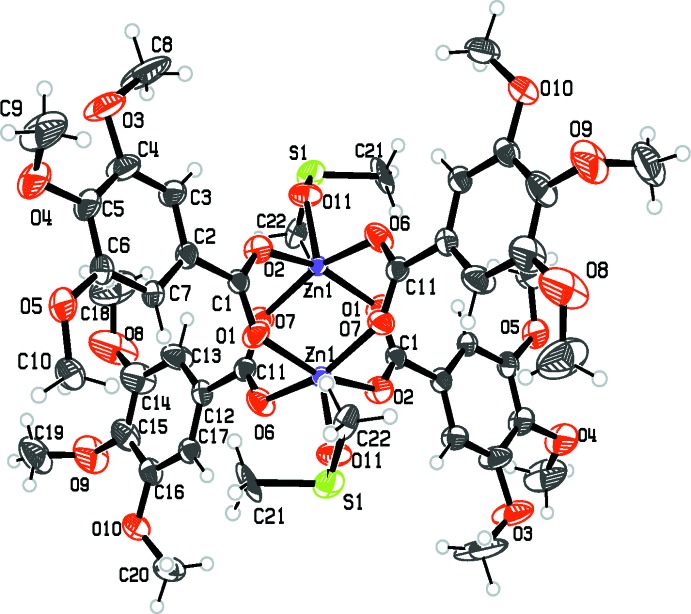
View of the molecular structure of bis(dimethyl sulfoxide-κO)tetrakis(µ2-3,4,5-trimethoxybenzoato-κ2O:O')dizinc; ellipsoids at 50% probability level (Symmetry codes: -x + 0.5, -y + 1.5,-z + 1).

## supplementary crystallographic information

### 1. Comment

In bis(dimethyl sulfoxide-κ*O*)tetrakis(µ_2_-3,4,5-trimethoxybenzoato-κ^2^*O*:*O*')dizinc the two Zn(ii) ions are embedded in a distorted square pyramidale environment. Four 3,4,5-trimethoxybenzoato-ligands are forming four µ_2_-O,*O*'-bridges between the two Zn-atoms. The coordination number is completed by a dmso solvent molecule in apical position. The Zn—O bond distances are determined in the range between 2.019 (3) Å and 2.042 (3) Å for the carboxylato-O atoms whereas the Zn—O bond distance to the coordinated solvent molecule is significantly shorter with 1.966 (3) Å. These findings are in accordance with the literature data for the so-called paddle-wheel structures formed by complexes of the type bis(dimethylsulfoxide-κ*O*)tetrakis(µ_2_-carboxylato-*O*,*O*')dizinc. In Pham *et al.* (2008) the Zn—O bond distances to carboxylato-O atoms are determined to in the mean 2.046 (3) Å whereas the Zn—O (dmso) bond distance is significantly shorter with 1.984 (3) Å. In Reger *et al.* (2011) Zn—O bond distances between 2.032 (2) and 2.051 (2) Å or of 197.2 (2) and 197.4 (2) Å are reported. In Tao *et al.* (2002) and Yang *et al.* (2005) these findings are confirmed furthermore: the corresponding Zn—O distances to the coordinated dmso are significantly shorter with 1.982 (3) Å or with 1.970 (2) and 1.981 (2) Å than those for the corresponding Zn—O bond lengths concerning the carboxylato groups (2.012 (2)–2.064 (3) Å). Changes in the coordination number of the central Zn(ii) ion like *e.g.* in Zevaco *et al.* (2007) influences significantly the Zn—O bond distances: for the Zn atom in a distorted tetrahedral environment Zn—O (carboxylato) bond lengths are found to be shorter with 1.933 (2) Å whereas they are determined to 2.073 (2) - 2.122 (2) Å for a Zn-atom in distorted octahedral coordination geometry. The structural features of bis(dimethyl sulfoxide-κ*O*)tetrakis(µ_2_-3,4,5-trimethoxybenzoato-κ^2^*O*:*O*')dizinc with the two Zn(ii) ions in a distorted square-pyramidal environment with their here reported bond lengths fit well within the in the literature reported related complexes.

### 2. Experimental

bis(dimethyl sulfoxide-κ*O*)tetrakis(µ_2_-3,4,5-trimethoxybenzoato-κ^2^*O*:*O*')dizinc is obtained from the reaction of 2.0 g (24.6 mmol) zinc oxide and 10.43 g (49.2 mmol) of 3,4,5-trimethoxybenzoic acid in water under refluxing for 4 h. The solvent is evaporated until formation of a white powder which was filtered off and dissolved in 150 ml DMSO at *ca* 120° C. The solution was filtered and allowed to cool down to RT. The solvent was evaporated slowly. Single crystals of bis(dimethyl sulfoxide-κ*O*)tetrakis(µ_2_-3,4,5-trimethoxybenzoato-κ^2^*O*:*O*')dizinc are isolated in 70% yield.

### 3. Refinement

The positions of all H atoms are calculated on geometrical positions according to the hybridization of the atoms they are bound to. The isotropic U values of the hydrogen atoms are refined group wise except for the H atoms which are located at the following disordered C atoms: C8, C8X, C18, C18X, C21X. *R*_int_ is with 0.12 relatively high as additional disorder of parts of the molecule plus some flexibility in the 12 methoxy substituents contribute to a decrease in reflection intensity for higher 2Θ-angles.

### Crystal data

**Table:**

[Zn_2_(C_10_H_11_O_5_)_4_(C_2_H_6_OS)_2_]	*F*(000) = 2352
*M**_r_* = 1131.75	*D*_x_ = 1.486 Mg m^−^^3^
Monoclinic, *C*2/*c*	Mo *K*α radiation, λ = 0.71073 Å
*a* = 18.854 (2) Å	Cell parameters from 6943 reflections
*b* = 13.937 (2) Å	θ = 2.4–26.0°
*c* = 19.249 (2) Å	µ = 1.11 mm^−^^1^
β = 90.082 (3)°	*T* = 200 K
*V* = 5058.0 (11) Å^3^	Quader, colourless
*Z* = 4	0.6 × 0.4 × 0.4 mm

### Data collection

**Table:**

Siemens SMART CCD 1000 diffractometer	6234 independent reflections
Radiation source: fine-focus sealed tube	4049 reflections with *I* > 2σ(*I*)
Graphite monochromator	*R*_int_ = 0.121
Detector resolution: 8 pixels mm^-1^	θ_max_ = 28.4°, θ_min_ = 1.8°
ω scan	*h* = −25→25
Absorption correction: multi-scan (*SADABS*; Bruker, 1997)	*k* = −18→18
*T*_min_ = 0.555, *T*_max_ = 1	*l* = −25→24
29984 measured reflections	

### Refinement

**Table:**

Refinement on *F*^2^	Primary atom site location: structure-invariant direct methods
Least-squares matrix: full	Secondary atom site location: difference Fourier map
*R*[*F*^2^ > 2σ(*F*^2^)] = 0.065	Hydrogen site location: inferred from neighbouring sites
*wR*(*F*^2^) = 0.164	H atoms treated by a mixture of independent and constrained refinement
*S* = 1.03	*w* = 1/[σ^2^(*F*_o_^2^) + (0.0719*P*)^2^ + 9.9666*P*] where *P* = (*F*_o_^2^ + 2*F*_c_^2^)/3
6234 reflections	(Δ/σ)_max_ < 0.001
378 parameters	Δρ_max_ = 0.64 e Å^−^^3^
19 restraints	Δρ_min_ = −0.79 e Å^−^^3^

### Special details

**Table:**

Experimental. Spectroscopic data: ^1^H{^31^P} NMR (dmso D_6_): δ = 7.18, s, 1H, CH(arom); 3.72, s, 6H, OCH_3_; 3.62, s, 3H, OCH_3_; ^13^C{^1^H} NMR (dmso D_3_): δ = 172.0; 152.7; 140.4; 130.4; 107.1; 60.6; 56.2; IR [cm^-1^]: 2995 (*m*); 2940 (*m*); 2837 (*m*); 1622 (*m*); 1577 (*s*); 1520 (*s*); 1464 (*m*); 1396 (*versus*); 1228 (*s*); 1127 (*versus*); 1000 (*m*); 786 (*s*); 759 (w); 733 (*m*)
Geometry. All e.s.d.'s (except the e.s.d. in the dihedral angle between two l.s. planes) are estimated using the full covariance matrix. The cell e.s.d.'s are taken into account individually in the estimation of e.s.d.'s in distances, angles and torsion angles; correlations between e.s.d.'s in cell parameters are only used when they are defined by crystal symmetry. An approximate (isotropic) treatment of cell e.s.d.'s is used for estimating e.s.d.'s involving l.s. planes.
Refinement. XRD measurements were performed on a Siemens *SMART* CCD 1000 diffractometer with monochromated Mo *K*α-irradiation collecting a full sphere of data in the θ–range from 1.82 to 28.36°. 1674 frames were collected with an irradiation time of 10 s per frame and ω–scan technique with Δω = 0.45°. The data were integrated with *SAINT* and corrected to Lorentz and polarization effects and a numerical adsorption correction with *SADABS* was applied. The structure was solved by direct methods and refined to an optimum *R*_1_ value with *SHELXL*. Visualization for evaluation was performed with XPMA and figures were created with *ORTEP*. Refinement of *F*^2^ against ALL reflections. The weighted *R*-factor *wR* and goodness of fit *S* are based on *F*^2^, conventional *R*-factors *R* are based on *F*, with *F* set to zero for negative *F*^2^. The threshold expression of *F*^2^ > σ(*F*^2^) is used only for calculating *R*-factors(gt) *etc*. and is not relevant to the choice of reflections for refinement. [tetrakis(µ_2_-3,4,5-trimethoxybenzoato-*O*,*O*')-bis(dimethylsulfoxide-O)-di-\ zinc(ii)] shows in its structure a 1:1 disorder at the following positions: C8, C18, C21, *c*22. The first two mentioned C atoms make part of two methoxy substituents whereas the last two mentioned C atoms represent a disorder in the coordinated dmso solvent molecule. Refinement of the disordered parts of the molecules has been performed using the 'same distance' restraint in order to resolve the disorders in a chemically correct manner. The data of the structure have been deposited at the CCDC with the reference number 865280.

### Fractional atomic coordinates and isotropic or equivalent isotropic displacement parameters (Å^2^)

**Table:**

	*x*	*y*	*z*	*U*_iso_*/*U*_eq_	Occ. (<1)
Zn1	0.24683 (2)	0.64467 (3)	0.49759 (2)	0.02114 (14)	
O1	0.28914 (16)	0.6564 (2)	0.59493 (14)	0.0376 (7)	
O2	0.29608 (16)	0.8154 (2)	0.59516 (14)	0.0397 (7)	
O3	0.4123 (3)	0.8959 (3)	0.82388 (19)	0.0800 (14)	
O4	0.4061 (2)	0.7307 (3)	0.89858 (18)	0.0728 (13)	
O5	0.35347 (18)	0.5704 (2)	0.84426 (15)	0.0482 (8)	
O6	0.14999 (15)	0.6644 (2)	0.53982 (17)	0.0421 (8)	
O7	0.15605 (15)	0.8236 (2)	0.54213 (16)	0.0390 (7)	
O8	−0.0668 (3)	0.9179 (3)	0.6706 (3)	0.121 (2)	
O9	−0.14597 (19)	0.7596 (3)	0.6733 (2)	0.0750 (13)	
O10	−0.09936 (16)	0.5933 (2)	0.62088 (17)	0.0476 (8)	
O11	0.24529 (16)	0.5055 (2)	0.48119 (17)	0.0442 (8)	
C1	0.30386 (19)	0.7354 (3)	0.6224 (2)	0.0265 (8)	
C2	0.3325 (2)	0.7336 (3)	0.6957 (2)	0.0298 (9)	
C3	0.3596 (2)	0.8172 (3)	0.7236 (2)	0.0400 (11)	
H3	0.3603	0.8734	0.6975	0.033 (8)*	
C4	0.3856 (3)	0.8163 (4)	0.7910 (2)	0.0520 (13)	
C5	0.3835 (3)	0.7325 (3)	0.8304 (2)	0.0468 (12)	
C6	0.3549 (2)	0.6487 (3)	0.8021 (2)	0.0352 (9)	
C7	0.3299 (2)	0.6497 (3)	0.73393 (19)	0.0290 (8)	
H7	0.3115	0.5941	0.7142	0.033 (8)*	
C8	0.416 (2)	0.9766 (12)	0.7778 (13)	0.18 (2)	0.5
H8A	0.4454	0.9605	0.7386	0.150*	0.5
H8B	0.4368	1.0302	0.8020	0.150*	0.5
H8C	0.3696	0.9932	0.7621	0.150*	0.5
C8X	0.3946 (18)	0.9896 (9)	0.7979 (12)	0.103 (9)	0.5
H8D	0.4173	1.0376	0.8259	0.150*	0.5
H8E	0.3441	0.9984	0.7996	0.150*	0.5
H8F	0.4104	0.9954	0.7507	0.150*	0.5
C9	0.4808 (4)	0.7422 (4)	0.9088 (3)	0.084 (2)	
H9A	0.5057	0.7195	0.8686	0.18 (2)*	
H9B	0.4955	0.7060	0.9488	0.18 (2)*	
H9C	0.4914	0.8089	0.9160	0.18 (2)*	
C10	0.3126 (3)	0.4904 (3)	0.8213 (3)	0.0568 (14)	
H10A	0.2643	0.5100	0.8144	0.050 (8)*	
H10B	0.3143	0.4406	0.8556	0.050 (8)*	
H10C	0.3316	0.4668	0.7783	0.050 (8)*	
C11	0.1254 (2)	0.7454 (3)	0.55252 (19)	0.0285 (8)	
C12	0.0531 (2)	0.7492 (3)	0.5848 (2)	0.0311 (8)	
C13	0.0284 (3)	0.8346 (3)	0.6115 (3)	0.0543 (14)	
H13	0.0555	0.8902	0.6081	0.055 (10)*	
C14	−0.0370 (3)	0.8365 (4)	0.6436 (4)	0.0678 (18)	
C15	−0.0792 (2)	0.7542 (4)	0.6456 (3)	0.0510 (13)	
C16	−0.0545 (2)	0.6694 (3)	0.6170 (2)	0.0346 (10)	
C17	0.0122 (2)	0.6669 (3)	0.5871 (2)	0.0303 (9)	
H17	0.0296	0.6099	0.5685	0.055 (10)*	
C18	−0.0156 (8)	0.9929 (11)	0.6886 (11)	0.105 (7)	0.5
H18A	0.0263	0.9640	0.7076	0.150*	0.5
H18B	−0.0362	1.0354	0.7222	0.150*	0.5
H18C	−0.0034	1.0285	0.6476	0.150*	0.5
C18X	−0.0416 (9)	1.0071 (7)	0.6374 (11)	0.125 (10)	0.5
H18D	−0.0647	1.0612	0.6583	0.150*	0.5
H18E	−0.0525	1.0053	0.5887	0.150*	0.5
H18F	0.0087	1.0127	0.6436	0.150*	0.5
C19	−0.1488 (4)	0.7388 (4)	0.7465 (3)	0.083 (2)	
H19A	−0.1422	0.6712	0.7537	0.16 (2)*	
H19B	−0.1941	0.7577	0.7646	0.16 (2)*	
H19C	−0.1120	0.7736	0.7701	0.16 (2)*	
C20	−0.0818 (3)	0.5103 (3)	0.5803 (3)	0.0539 (13)	
H20A	−0.0760	0.5286	0.5326	0.080 (11)*	
H20B	−0.1193	0.4639	0.5839	0.080 (11)*	
H20C	−0.0384	0.4828	0.5973	0.080 (11)*	
S1	0.28196 (19)	0.41693 (19)	0.50269 (16)	0.0463 (8)	0.5
C21	0.2543 (11)	0.4155 (12)	0.5903 (6)	0.063 (4)	0.5
H21A	0.2050	0.3983	0.5925	0.41 (13)*	0.5
H21B	0.2609	0.4779	0.6102	0.41 (13)*	0.5
H21C	0.2818	0.3694	0.6157	0.41 (13)*	0.5
C22	0.3713 (4)	0.4506 (6)	0.5154 (4)	0.036 (2)	0.5
H22A	0.3945	0.4560	0.4712	0.048 (15)*	0.5
H22B	0.3949	0.4029	0.5430	0.048 (15)*	0.5
H22C	0.3731	0.5113	0.5389	0.048 (15)*	0.5
S1X	0.28535 (16)	0.4397 (2)	0.5380 (2)	0.0522 (8)	0.5
C21X	0.2680 (8)	0.3248 (7)	0.5050 (8)	0.106 (6)	0.5
H21D	0.2948	0.3152	0.4632	0.200*	0.5
H21E	0.2183	0.3189	0.4948	0.200*	0.5
H21F	0.2814	0.2775	0.5387	0.200*	0.5
C22X	0.2319 (13)	0.4262 (19)	0.6110 (9)	0.108 (8)	0.5
H22D	0.2329	0.4843	0.6379	0.200*	0.5
H22E	0.2493	0.3740	0.6388	0.200*	0.5
H22F	0.1841	0.4129	0.5967	0.200*	0.5

### Atomic displacement parameters (Å^2^)

**Table:**

	*U*^11^	*U*^22^	*U*^33^	*U*^12^	*U*^13^	*U*^23^
Zn1	0.0257 (2)	0.0168 (2)	0.0209 (2)	−0.00103 (18)	−0.00125 (15)	0.00041 (18)
O1	0.0544 (19)	0.0325 (16)	0.0259 (14)	−0.0037 (13)	−0.0117 (13)	0.0000 (12)
O2	0.0537 (19)	0.0335 (16)	0.0318 (16)	−0.0030 (14)	−0.0144 (14)	0.0102 (13)
O3	0.135 (4)	0.051 (2)	0.053 (2)	−0.034 (2)	−0.049 (2)	0.0011 (19)
O4	0.106 (3)	0.081 (3)	0.0316 (19)	−0.026 (2)	−0.030 (2)	0.0061 (17)
O5	0.070 (2)	0.0471 (19)	0.0270 (16)	−0.0041 (16)	−0.0104 (15)	0.0134 (14)
O6	0.0342 (16)	0.0344 (17)	0.058 (2)	0.0025 (13)	0.0159 (15)	0.0036 (14)
O7	0.0322 (16)	0.0381 (16)	0.0469 (18)	−0.0089 (13)	0.0113 (14)	−0.0097 (14)
O8	0.084 (3)	0.058 (3)	0.221 (6)	−0.008 (2)	0.099 (4)	−0.045 (3)
O9	0.0320 (18)	0.093 (3)	0.100 (3)	0.0028 (19)	0.027 (2)	−0.003 (2)
O10	0.0393 (17)	0.052 (2)	0.051 (2)	−0.0156 (15)	0.0073 (15)	0.0000 (16)
O11	0.0460 (18)	0.0218 (15)	0.065 (2)	−0.0007 (13)	−0.0023 (16)	−0.0019 (14)
C1	0.0228 (18)	0.032 (2)	0.0244 (19)	−0.0026 (15)	0.0003 (14)	0.0024 (16)
C2	0.029 (2)	0.038 (2)	0.0218 (19)	−0.0023 (16)	−0.0043 (15)	0.0040 (16)
C3	0.054 (3)	0.037 (2)	0.029 (2)	−0.010 (2)	−0.013 (2)	0.0055 (19)
C4	0.075 (4)	0.040 (3)	0.041 (3)	−0.017 (2)	−0.027 (3)	0.002 (2)
C5	0.065 (3)	0.051 (3)	0.024 (2)	−0.010 (2)	−0.015 (2)	0.0036 (19)
C6	0.042 (2)	0.039 (2)	0.024 (2)	−0.002 (2)	−0.0019 (17)	0.0061 (18)
C7	0.033 (2)	0.031 (2)	0.0226 (18)	−0.0009 (17)	−0.0007 (15)	0.0025 (17)
C8	0.40 (5)	0.070 (13)	0.057 (15)	−0.13 (2)	−0.075 (19)	0.016 (10)
C8X	0.21 (2)	0.045 (9)	0.053 (12)	−0.041 (10)	−0.088 (15)	−0.001 (7)
C9	0.107 (6)	0.076 (5)	0.067 (4)	−0.008 (4)	−0.060 (4)	0.001 (3)
C10	0.090 (4)	0.043 (3)	0.038 (3)	−0.019 (3)	−0.003 (3)	0.017 (2)
C11	0.0263 (18)	0.034 (2)	0.0251 (19)	−0.0007 (18)	−0.0027 (15)	−0.0019 (18)
C12	0.0296 (19)	0.037 (2)	0.0272 (19)	0.0007 (19)	0.0032 (15)	0.0012 (18)
C13	0.037 (3)	0.043 (3)	0.083 (4)	−0.009 (2)	0.024 (3)	−0.013 (3)
C14	0.047 (3)	0.052 (3)	0.105 (5)	0.004 (2)	0.037 (3)	−0.019 (3)
C15	0.028 (2)	0.061 (3)	0.064 (3)	0.001 (2)	0.015 (2)	0.004 (3)
C16	0.024 (2)	0.046 (3)	0.034 (2)	−0.0021 (17)	−0.0006 (17)	0.0056 (19)
C17	0.028 (2)	0.037 (2)	0.026 (2)	−0.0003 (16)	−0.0024 (16)	0.0027 (17)
C18	0.073 (11)	0.087 (12)	0.153 (18)	0.007 (9)	0.007 (11)	−0.064 (12)
C18X	0.098 (13)	0.027 (6)	0.25 (3)	−0.001 (7)	0.113 (16)	−0.038 (11)
C19	0.080 (5)	0.068 (4)	0.101 (6)	−0.004 (3)	0.057 (4)	0.003 (4)
C20	0.049 (3)	0.052 (3)	0.061 (3)	−0.022 (2)	−0.004 (3)	0.002 (3)
S1	0.0740 (19)	0.0196 (13)	0.0452 (17)	0.0047 (12)	0.0011 (15)	−0.0029 (11)
C21	0.092 (11)	0.051 (7)	0.047 (9)	−0.008 (7)	0.032 (7)	0.033 (7)
C22	0.047 (5)	0.038 (5)	0.024 (4)	0.020 (4)	−0.007 (4)	−0.001 (3)
S1X	0.0506 (15)	0.0197 (13)	0.086 (3)	0.0076 (11)	0.0008 (19)	0.0125 (16)
C21X	0.148 (15)	0.018 (5)	0.152 (15)	0.011 (7)	0.004 (12)	−0.004 (7)
C22X	0.14 (2)	0.142 (17)	0.047 (10)	0.008 (14)	0.040 (10)	0.048 (10)

### Geometric parameters (Å, º)

**Table:**

Zn1—O11	1.966 (3)	C9—H9A	0.9600
Zn1—O6	2.019 (3)	C9—H9B	0.9600
Zn1—O7^i^	2.034 (3)	C9—H9C	0.9600
Zn1—O2^i^	2.037 (3)	C10—H10A	0.9600
Zn1—O1	2.042 (3)	C10—H10B	0.9600
Zn1—Zn1^i^	2.9399 (9)	C10—H10C	0.9600
O1—C1	1.252 (4)	C11—C12	1.501 (5)
O2—C1	1.240 (4)	C12—C13	1.378 (6)
O2—Zn1^i^	2.037 (3)	C12—C17	1.382 (5)
O3—C4	1.372 (6)	C13—C14	1.380 (6)
O3—C8	1.435 (9)	C13—H13	0.9300
O3—C8X	1.439 (9)	C14—C15	1.396 (7)
O4—C5	1.380 (5)	C15—C16	1.384 (6)
O4—C9	1.431 (6)	C16—C17	1.386 (5)
O5—C6	1.360 (5)	C17—H17	0.9300
O5—C10	1.425 (5)	C18—H18A	0.9600
O6—C11	1.244 (5)	C18—H18B	0.9600
O7—C11	1.249 (5)	C18—H18C	0.9600
O7—Zn1^i^	2.034 (3)	C18X—H18D	0.9600
O8—C14	1.368 (6)	C18X—H18E	0.9600
O8—C18	1.464 (8)	C18X—H18F	0.9600
O8—C18X	1.476 (8)	C19—H19A	0.9600
O9—C15	1.371 (5)	C19—H19B	0.9600
O9—C19	1.439 (6)	C19—H19C	0.9600
O10—C16	1.358 (5)	C20—H20A	0.9600
O10—C20	1.435 (5)	C20—H20B	0.9600
O11—S1	1.474 (4)	C20—H20C	0.9600
O11—S1X	1.614 (4)	S1—C21	1.765 (9)
C1—C2	1.511 (5)	S1—C22	1.766 (7)
C2—C3	1.381 (6)	C21—H21A	0.9600
C2—C7	1.382 (5)	C21—H21B	0.9600
C3—C4	1.388 (6)	C21—H21C	0.9600
C3—H3	0.9300	C22—H22A	0.9600
C4—C5	1.392 (6)	C22—H22B	0.9600
C5—C6	1.397 (6)	C22—H22C	0.9600
C6—C7	1.394 (5)	S1X—C22X	1.741 (9)
C7—H7	0.9300	S1X—C21X	1.753 (8)
C8—H8A	0.9600	C21X—H21D	0.9600
C8—H8B	0.9600	C21X—H21E	0.9600
C8—H8C	0.9600	C21X—H21F	0.9600
C8X—H8D	0.9600	C22X—H22D	0.9600
C8X—H8E	0.9600	C22X—H22E	0.9600
C8X—H8F	0.9600	C22X—H22F	0.9600
			
O11—Zn1—O6	100.72 (12)	O6—C11—C12	116.9 (4)
O11—Zn1—O7^i^	99.65 (12)	O7—C11—C12	117.1 (4)
O6—Zn1—O7^i^	159.55 (13)	C13—C12—C17	121.1 (4)
O11—Zn1—O2^i^	97.09 (13)	C13—C12—C11	119.5 (4)
O6—Zn1—O2^i^	87.56 (13)	C17—C12—C11	119.3 (4)
O7^i^—Zn1—O2^i^	88.14 (13)	C12—C13—C14	119.1 (4)
O11—Zn1—O1	103.43 (12)	C12—C13—H13	120.5
O6—Zn1—O1	88.39 (13)	C14—C13—H13	120.5
O7^i^—Zn1—O1	88.67 (12)	O8—C14—C13	123.6 (5)
O2^i^—Zn1—O1	159.48 (12)	O8—C14—C15	115.9 (4)
O11—Zn1—Zn1^i^	172.42 (10)	C13—C14—C15	120.4 (5)
O6—Zn1—Zn1^i^	83.57 (8)	O9—C15—C16	120.7 (4)
O7^i^—Zn1—Zn1^i^	75.99 (9)	O9—C15—C14	119.3 (4)
O2^i^—Zn1—Zn1^i^	76.73 (9)	C16—C15—C14	119.9 (4)
O1—Zn1—Zn1^i^	82.82 (8)	O10—C16—C15	115.8 (4)
C1—O1—Zn1	123.0 (3)	O10—C16—C17	124.7 (4)
C1—O2—Zn1^i^	131.5 (3)	C15—C16—C17	119.5 (4)
C4—O3—C8	111.7 (11)	C12—C17—C16	119.9 (4)
C4—O3—C8X	119.2 (9)	C12—C17—H17	120.0
C5—O4—C9	115.6 (5)	C16—C17—H17	120.0
C6—O5—C10	116.9 (3)	O8—C18—H18A	109.5
C11—O6—Zn1	122.7 (3)	O8—C18—H18B	109.5
C11—O7—Zn1^i^	131.8 (3)	H18A—C18—H18B	109.5
C14—O8—C18	114.3 (8)	O8—C18—H18C	109.5
C14—O8—C18X	113.6 (7)	H18A—C18—H18C	109.5
C15—O9—C19	113.9 (5)	H18B—C18—H18C	109.5
C16—O10—C20	117.1 (3)	O8—C18X—H18D	109.5
S1—O11—Zn1	140.8 (2)	O8—C18X—H18E	109.5
S1X—O11—Zn1	116.4 (2)	H18D—C18X—H18E	109.5
O2—C1—O1	125.8 (4)	O8—C18X—H18F	109.5
O2—C1—C2	116.9 (3)	H18D—C18X—H18F	109.5
O1—C1—C2	117.3 (3)	H18E—C18X—H18F	109.5
C3—C2—C7	121.4 (4)	O9—C19—H19A	109.5
C3—C2—C1	118.7 (3)	O9—C19—H19B	109.5
C7—C2—C1	119.9 (3)	H19A—C19—H19B	109.5
C2—C3—C4	119.0 (4)	O9—C19—H19C	109.5
C2—C3—H3	120.5	H19A—C19—H19C	109.5
C4—C3—H3	120.5	H19B—C19—H19C	109.5
O3—C4—C3	123.6 (4)	O10—C20—H20A	109.5
O3—C4—C5	116.0 (4)	O10—C20—H20B	109.5
C3—C4—C5	120.4 (4)	H20A—C20—H20B	109.5
O4—C5—C4	121.6 (4)	O10—C20—H20C	109.5
O4—C5—C6	118.3 (4)	H20A—C20—H20C	109.5
C4—C5—C6	120.0 (4)	H20B—C20—H20C	109.5
O5—C6—C7	124.3 (4)	O11—S1—C21	98.0 (6)
O5—C6—C5	116.5 (4)	O11—S1—C22	105.3 (3)
C7—C6—C5	119.2 (4)	C21—S1—C22	98.9 (7)
C2—C7—C6	119.8 (4)	S1—C21—H21A	109.5
C2—C7—H7	120.1	S1—C21—H21B	109.5
C6—C7—H7	120.1	H21A—C21—H21B	109.5
O3—C8—H8A	109.5	S1—C21—H21C	109.5
O3—C8—H8B	109.5	H21A—C21—H21C	109.5
H8A—C8—H8B	109.5	H21B—C21—H21C	109.5
O3—C8—H8C	109.5	S1—C22—H22A	109.5
H8A—C8—H8C	109.5	S1—C22—H22B	109.5
H8B—C8—H8C	109.5	H22A—C22—H22B	109.5
O3—C8X—H8D	109.5	S1—C22—H22C	109.5
O3—C8X—H8E	109.5	H22A—C22—H22C	109.5
H8D—C8X—H8E	109.5	H22B—C22—H22C	109.5
O3—C8X—H8F	109.5	O11—S1X—C22X	109.7 (9)
H8D—C8X—H8F	109.5	O11—S1X—C21X	100.7 (5)
H8E—C8X—H8F	109.5	C22X—S1X—C21X	95.0 (10)
O4—C9—H9A	109.5	S1X—C21X—H21D	109.5
O4—C9—H9B	109.5	S1X—C21X—H21E	109.5
H9A—C9—H9B	109.5	H21D—C21X—H21E	109.5
O4—C9—H9C	109.5	S1X—C21X—H21F	109.5
H9A—C9—H9C	109.5	H21D—C21X—H21F	109.5
H9B—C9—H9C	109.5	H21E—C21X—H21F	109.5
O5—C10—H10A	109.5	S1X—C22X—H22D	109.5
O5—C10—H10B	109.5	S1X—C22X—H22E	109.5
H10A—C10—H10B	109.5	H22D—C22X—H22E	109.5
O5—C10—H10C	109.5	S1X—C22X—H22F	109.5
H10A—C10—H10C	109.5	H22D—C22X—H22F	109.5
H10B—C10—H10C	109.5	H22E—C22X—H22F	109.5
O6—C11—O7	126.0 (4)		

Symmetry code: (i) −*x*+1/2, −*y*+3/2, −*z*+1.
